# Erasure conversion for fault-tolerant quantum computing in alkaline earth Rydberg atom arrays

**DOI:** 10.1038/s41467-022-32094-6

**Published:** 2022-08-09

**Authors:** Yue Wu, Shimon Kolkowitz, Shruti Puri, Jeff D. Thompson

**Affiliations:** 1 Department of Computer Science, Yale University, New Haven, CT 06520 USA; 2 Department of Physics, University of Wisconsin-Madison, Madison, WI 53706 USA; 3 Department of Applied Physics, Yale University, New Haven, CT 06520 USA; 4 Department of Electrical and Computer Engineering, Princeton University, Princeton, NJ 08544 USA

**Keywords:** Qubits, Quantum information, Atomic and molecular physics

## Abstract

Executing quantum algorithms on error-corrected logical qubits is a critical step for scalable quantum computing, but the requisite numbers of qubits and physical error rates are demanding for current experimental hardware. Recently, the development of error correcting codes tailored to particular physical noise models has helped relax these requirements. In this work, we propose a qubit encoding and gate protocol for ^171^Yb neutral atom qubits that converts the dominant physical errors into erasures, that is, errors in known locations. The key idea is to encode qubits in a metastable electronic level, such that gate errors predominantly result in transitions to disjoint subspaces whose populations can be continuously monitored via fluorescence. We estimate that 98% of errors can be converted into erasures. We quantify the benefit of this approach via circuit-level simulations of the surface code, finding a threshold increase from 0.937% to 4.15%. We also observe a larger code distance near the threshold, leading to a faster decrease in the logical error rate for the same number of physical qubits, which is important for near-term implementations. Erasure conversion should benefit any error correcting code, and may also be applied to design new gates and encodings in other qubit platforms.

## Abstract (web summary)

In quantum computing, realistic error models can allow tailored correction schemes for specific platforms. Here, while considering the case of qubits encoded in metastable electronic levels of atomic arrays, the authors propose a way to convert a large fraction of occurring errors into detectable leakages, or erasure errors, which are vastly easier to correct.

## Introduction

Scalable, universal quantum computers have the potential to outperform classical computers for a range of tasks^1^. However, the inherent fragility of quantum states and the finite fidelity of physical qubit operations make errors unavoidable in any quantum computation. Quantum error correction^2–4^ allows multiple physical qubits to represent a single logical qubit, such that the correct logical state can be recovered even in the presence of errors on the underlying physical qubits and gate operations.

If the logical qubit operations are implemented in a fault-tolerant manner that prevents the proliferation of correlated errors, the logical error rate can be suppressed arbitrarily so long as the error probability during each operation is below a threshold^5,6^. Fault-tolerant protocols for error correction and logical qubit manipulation have recently been experimentally demonstrated in several platforms^7–10^.

The threshold error rate depends on the choice of error correcting code and the nature of the noise in the physical qubit. While many codes have been studied in the context of the abstract model of depolarizing noise arising from the action of random Pauli operators on the qubit, the realistic error model for a given qubit platform is often more complex, which presents both opportunities and challenges. For example, qubits encoded in cat-codes in superconducting resonators can have strongly biased noise^11^, leading to significantly higher thresholds^12,13^ given suitable bias-preserving gate operations for fault-tolerant syndrome extraction^14^. The realization of biased noise models and bias-preserving gates for Rydberg atom arrays has also been discussed^15^. On the other hand, many qubits also exhibit some level of leakage outside of the computational space^6,16^, which requires extra gates in the form of leakage-reducing units, decreasing the threshold^17^.

Another type of error is an erasure, or detectable leakage, which denotes an error at a known location. Erasures are significantly easier to correct than depolarizing errors in both classical^18^ and quantum^3,19^ settings. For example, a four-qubit quantum code is sufficient to correct a single erasure error^19^, and the surface code threshold under the erasure channel approaches 50% (with perfect syndrome measurements), saturating the bound imposed by the no-cloning theorem^20^. Erasure errors arise naturally in photonic qubits: if a qubit is encoded in the polarization, or path, of a single photon, then the absence of a photon detection signals an erasure, allowing efficient error correction for quantum communication^21^ and linear optics quantum computing^22,23^. However, techniques for detecting the locations of errors in matter-based qubits have not been extensively studied.

In this work, we present an approach to fault-tolerant quantum computing in Rydberg atom arrays^24–26^ based on converting a large fraction of naturally occurring errors into erasures. Our work has two key components. First, we present a physical model of qubits encoded in a particular atomic species, ^171^Yb^27–29^, that enables erasure conversion without additional gates or ancilla qubits. By encoding qubits in the hyperfine states of a metastable electronic level, the vast majority of errors (i.e., decays from the Rydberg state that is used to implement two-qubit gates) result in transitions out of the computational subspace into levels whose population can be continuously monitored using cycling transitions that do not disturb the qubit levels (the use of a metastable state to certify the absence of certain errors was also recently proposed for trapped ion qubits^30^). As a result, the location of these errors is revealed, converting them into erasures. We estimate a fraction *R*_*e*_ = 0.98 of all errors can be detected this way. Second, we quantify the benefit of erasure conversion at the circuit level, using simulations of the surface code. We find that the predicted level of erasure conversion results in a significantly higher threshold, *p*_th_ = 4.15%, compared to the case of pure depolarizing errors (*p*_th_ = 0.937%). Finally, we find a faster reduction in the logical error rate immediately below the threshold.

## Results

### Erasure conversion in ^171^Yb qubits

**Fig. 1:**
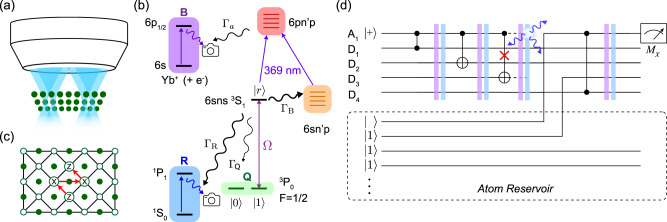
Overview of a fault-tolerant neutral atom quantum computer using erasure conversion. **a** Schematic of a neutral atom quantum computer, with a plane of atoms under a microscope objective used to image fluorescence and project trapping and control fields. **b** The physical qubits are individual ^171^Yb atoms. The qubit states are encoded in the metastable 6*s*6*p* ^3^P_0_*F* = 1/2 level (subspace Q), and two-qubit gates are performed via the Rydberg state $\left|r\right\rangle$, which is accessed through a single-photon transition (*λ* = 302 nm) with Rabi frequency Ω. The dominant errors during gates are decays from $\left|r\right\rangle$ with a total rate Γ = Γ_*B*_ + Γ_*R*_ + Γ_*Q*_. Only a small fraction Γ_*Q*_/Γ ≈ 0.05 return to the qubit subspace, while the remaining decays are either blackbody (BBR) transitions to nearby Rydberg states (Γ_*B*_/Γ ≈ 0.61) or radiative decay to the ground state 6*s*^2^ ^1^*S*_0_ (Γ_*R*_/Γ ≈ 0.34). At the end of a gate, these events can be detected and converted into erasure errors by detecting fluorescence from ground state atoms (subspace R), or ionizing any remaining Rydberg population via autoionization, and collecting fluorescence on the Yb^+^ transition (subspace B). **c** A patch of the XZZX surface code studied in this work, showing data qubits (open circles), ancilla qubits (filled circles) and stabilizer operations, performed in the order indicated by the arrows. **d** Quantum circuit representing a measurement of a stabilizer on data qubits *D*_1_ − *D*_4_ using ancilla *A*_1_ with interleaved erasure conversion steps. Erasure detection is applied after each gate, and erased atoms are replaced from a reservoir as needed using a moveable optical tweezer. It is strictly only necessary to replace the atom that was detected to have left the subspace, but replacing both protects against the possibility of undetected leakage on the second atom.

In a neutral atom quantum computer, an array of atomic qubits are trapped, manipulated, and detected using light projected through a microscope objective (Fig. 1a). A variety of atomic species have been explored, but in this work, we consider ^171^Yb^28,29^, with the qubit encoded in the *F* = 1/2 6*s*6*p* ^3^*P*_0_ (Fig. 1b) level. This is commonly used as the upper level of optical atomic clocks^31^, and is metastable with a lifetime of *τ* ≈ 20 s. We define the qubit states as $\left|1\right\rangle \equiv \left|{m}_{F}=1/2\right\rangle$ and $\left|0\right\rangle \equiv \left|{m}_{F}={-}1/2\right\rangle$. State preparation, measurement and single-qubit rotations can be performed in a manner similar to existing neutral atom qubits, and a detailed prescription is presented in Supplementary Note 1.

To perform two-qubit gates, the state $\left|1\right\rangle$ is coupled to a Rydberg state $\left|r\right\rangle$ with Rabi frequency Ω. For concreteness, we consider the 6*s*75*s* ^3^*S*_1_ state with $\left|F,{m}_{F}\right\rangle=\left|3/2,\,3/2\right\rangle$^32^. Selective coupling of $\left|1\right\rangle$ to $\left|r\right\rangle$ can be achieved by using a circularly polarized laser and a large magnetic field to detune the transition from $\left|0\right\rangle$ to the *m*_*F*_ = 1/2 Rydberg state^28^.

The resulting three level system $\{\left|0\right\rangle,\;\left|1\right\rangle,\;\left|r\right\rangle \}$ is analogous to hyperfine qubits encoded in alkali atoms, for which numerous gate protocols have been proposed and demonstrated^24,25,33–37^. These gates are based on the Rydberg blockade: the van der Waals interaction *V*_*rr*_(*x*) = *C*_6_/*x*^6^ between a pair of Rydberg atoms separated by *x* prevents their simultaneous excitation to $\left|r\right\rangle$ if *V*_*rr*_(*x*) ≫ Ω. The gate duration is of order *t*_*g*_ ≈ 2*π*/Ω ≫ 2*π*/*V*_*rr*_, and during this time, the Rydberg state can decay with probability $p=\left\langle {P}_{r}\right\rangle {{\Gamma }}{t}_{g}$, where $\left\langle {P}_{r}\right\rangle \;\approx\; 1/2$ is the average population in $\left|r\right\rangle$ during the gate, and Γ is the total decay rate from $\left|r\right\rangle$. This is the fundamental limitation to the fidelity of Rydberg gates^26^. It can be suppressed by increasing Ω (up to the limit imposed by *V*_*rr*_), but in practice, Ω is often constrained by the available laser power. We note that the Yb ^3^*S*_1_ series has similar interaction strength^28,38^ and lifetime^32^ to Rydberg series in alkali atoms.

The state $\left|r\right\rangle$ can decay via radiative decay to low-lying states (RD), or via blackbody-induced transitions to nearby Rydberg states (BBR)^26^. Crucially, a large fraction of RD events do not reach the metastable qubit subspace *Q*, but instead go to the true atomic ground state 6*s*^2^ ^1^*S*_0_ (with suitable repumping of the other metastable state, 6*s*6*p* ^3^*P*_2_). For an *n* = 75 ^3^*S*_1_ Rydberg state, we estimate that 61% of decays are BBR, 34% are RD to the ground state, and only 5% are RD to the qubit subspace (see Supplementary Note 2). Therefore, a total of 95% of all decays leave the qubit in disjoint subspaces, whose population can be detected efficiently, converting these errors into erasures. The remaining 5% can only cause errors in the computational space—there is no possibility for leakage, as the *Q* subspace has only two sublevels.

Decays to states outside of *Q* can be detected using fluorescence on closed cycling transitions that do not disturb atoms in *Q*. Population in the ^1^*S*_0_ level can be efficiently detected using fluorescence on the ^1^*P*_1_ transition at 399 nm^39,40^ (subspace *R* in Fig. 1c). This transition is highly cyclic, with a branching ratio of ≈1 × 10^−7^ back into *Q*^41^. Population remaining in Rydberg states at the end of a gate can be converted into Yb^+^ ions by autoionization on the 6*s* → 6*p*_1/2_ Yb^+^ transition at 369 nm^38^. The resulting slow-moving Yb^+^ ions can be detected using fluorescence on the same Yb^+^ transition, as has been previously demonstrated for ensembles of Sr^+^ ions in ultracold strontium gases^42^ (subspace *B* in Fig. 1c). As the ions can be removed after each erasure detection round with a small electric field, this approach also eliminates correlated errors from leakage to long-lived Rydberg states^43^. We estimate that site-resolved detection of atoms in ^1^*S*_0_ with a fidelity *F* > 0.999^44^, and Yb^+^ ions with a fidelity *F* > 0.99, can be achieved in a 10 μs imaging period (see Supplementary Note 3). We note that two nearby ions created in the same cycle will likely not be detected because of mutual repulsion, but this occurs with a very small probability relative to other errors, as discussed below.

**Fig. 2:**
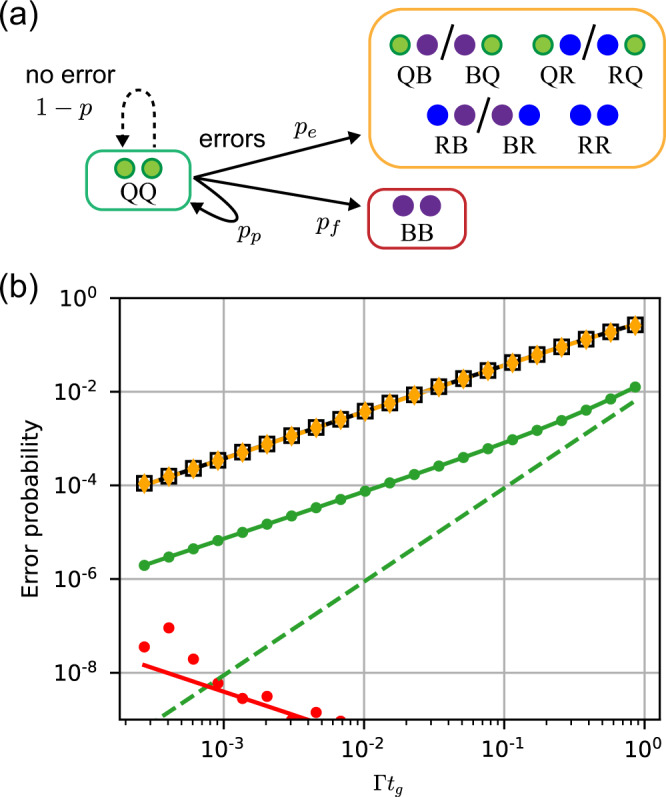
Gate error model and simulated performance. **a** Possible atomic states at the end of a two-qubit gate. The configurations grouped in the yellow box are detectable erasure errors; red, undetectable errors; and green, the computational space. **b** Gate error as a function of the gate duration *t*_*g*_. The average gate infidelity $1-{{{{{{{\mathcal{F}}}}}}}}$ (black squares) is dominated by detectable erasures with probability *p*_*e*_ (orange points). The infidelity conditioned on not detecting an erasure, $1-{{{{{{{{\mathcal{F}}}}}}}}}_{\bar{e}}$ (green points) is about 50 times smaller. This reflects decays to *Q* with probability *p*_*p*_, and a no-jump evolution contribution (green dashed line). The probability *p*_*f*_ of undetectable leakage (red points) is very small. The lines are analytic estimates of each quantity, while the symbols are numerical simulations. Both assume *V*_*rr*_/Γ = 10^6^, and Ω is varied along the horizontal axis.

We divide the total spontaneous emission probability, *p*, into three classes depending on the final state of the atoms (Fig. 2a). The first outcome is stated corresponding to detectable erasures (BQ/QB, RQ/QR, RB/BR, and RR), with probability *p*_*e*_. The second is the creation of two ions (BB), which cannot be detected, occurring with probability *p*_*f*_. The third outcome is a return to the qubit subspace (QQ), with probability *p*_*p*_, which results in an error on the qubits within the computational space.

The value of *p* and its decomposition depends on the specific Rydberg gate protocol. We study a particular example, the symmetric CZ gate from ref. 35, using a combination of analytic and numerical techniques, detailed in Supplementary Note 4 and summarized in Fig. 2b. The probability of a detectable erasure, *p*_*e*_, is almost identical to the average gate infidelity $1-{{{{{{{\mathcal{F}}}}}}}}$, indicating that the vast majority of errors are of this type. We infer *p*_*p*_ from the fidelity conditioned on not detecting an erasure, ${{{{{{{{\mathcal{F}}}}}}}}}_{\bar{e}}$, as ${p}_{p}=1-{{{{{{{{\mathcal{F}}}}}}}}}_{\bar{e}}$, and find *p*_*p*_ ≈ *p*_*e*_/50. Non-detectable leakage (*BB*) is strongly suppressed by the Rydberg blockade, and we find *p*_*f*_ < 10^−4^ × *p*_*e*_ over the relevant parameter range. Since decays occur preferentially from $\left|1\right\rangle$, continuously monitoring for erasures introduces an additional probability of gate error from non-Hermitian no-jump evolution^45^, proportional to ${p}_{e}^{2}$, which is insignificant for *p*_*e*_ < 0.1 (see Methods).

We conclude that this approach effectively converts a fraction *R*_*e*_ = *p*_*e*_/(*p*_*e*_ + *p*_*p*_) = 0.98 of all spontaneous decay errors into erasures. This is a larger fraction than would be naïvely predicted from the branching ratio into the qubit subspace, 1 − Γ_*Q*_/Γ = 0.95, because decays to *Q* in the middle of the gate result in re-excitation to $\left|r\right\rangle$ with a high probability, triggering an erasure detection. This value is in agreement with an analytic estimate (Supplementary Note 4).

### Surface code simulations

We now study the performance of an error correcting code with erasure conversion using circuit-level simulations. We consider the planar XZZX surface code^46^, which has been studied in the context of biased noise, and performs identically to the standard surface code for the case of unbiased noise. We implement Monte Carlo simulations of errors in a *d* × *d* array of data qubits to implement a code with distance *d*, and estimate the logical failure rate after *d* rounds of measurements.

In the simulation, each two-qubit gate experiences either a Pauli error with probability *p*_*p*_ = *p*(1 − *R*_*e*_), or an erasure with probability *p*_*e*_ = *pR*_*e*_. The Pauli errors are drawn uniformly at random from the set {*I*, *X*, *Y*, *Z*}^⊗2^\{*I* ⊗ *I*}, each with probability *p*_*p*_/15. Following a two-qubit gate in which an erasure error occurs, both atoms are placed in the mixed state *I*/2, which is modeled in the simulations by applying a Pauli error chosen uniformly at random from {*I*, *X*, *Y*, *Z*}^⊗2^^47^ (in the experiment, the replaced atoms can be in any state, since the subsequent stabilizer measurements and decoding are equivalent to a depolarizing error). We do not consider single-qubit gate errors or ancilla initialization or measurement errors at this stage.

The syndrome measurement results, together with the locations of the erasure errors, are decoded with weighted Union Find (UF) decoder^48,49^ to determine whether the error is correctable or leads to a logical failure. The UF decoder is optimal for pure erasure errors^50^, and performs comparably to conventional matching decoders for Pauli errors, but is considerably faster^48,49^.

**Fig. 3:**
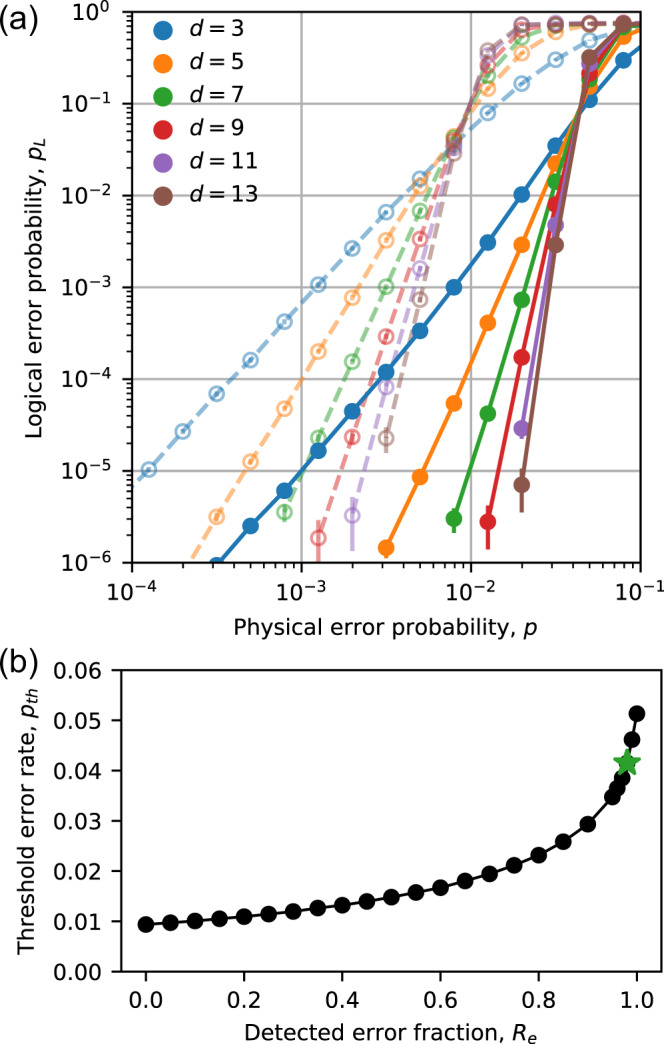
Circuit-level error thresholds in the presence of erasure errors. **a** Scaling of the logical error rate with the physical qubit error rate *p* in the case of pure computational errors (*R*_*e*_ = 0, open circles, dashed lines) and in the case of a high conversion to erasure errors, *R*_*e*_ = 0.98 (filled circles, solid lines). The error thresholds are *p*_th_ = 0.937(4)% and *p*_th_ = 4.15(2)%, respectively, determined from the crossing of *d* = 11 and *d* = 15. The error bars indicate the 95% confidence interval in *p*_*L*_, estimated from the number of trials in the Monte Carlo simulation. **b** *p*_*th*_ as a function of *R*_*e*_ (The green star highlights *R*_*e*_ = 0.98).

In Fig. 3a, we present the simulation results for *R*_*e*_ = 0 and *R*_*e*_ = 0.98. The former corresponds to pure Pauli errors, while the latter corresponds to the level of erasure conversion anticipated in ^171^Yb. The logical errors are significantly reduced in the latter case. The fault-tolerance threshold, defined as the physical error rate where the logical error rate decreases with increasing *d*, increases by a factor of 4.4, from *p*_th_ = 0.937% to *p*_th_ = 4.15%. In Fig. 3b, we plot the threshold as a function of *R*_*e*_. It reaches 5.13% when *R*_*e*_ = 1. The smooth increase of the threshold with *R*_*e*_ is qualitatively consistent with previous studies of the surface code performance with mixed erasure and Pauli errors^20,48,51^.

In addition to increasing the threshold, the high fraction of erasure errors also results in a faster decrease in the logical error rate below the threshold. Below the threshold, *p*_*L*_ can be approximated by *Ap*^*ν*^, where the exponent *ν* is the number of errors needed to cause a logical failure. A larger value of *ν* results in a faster suppression of logical errors below the threshold, and better code performance for a fixed number of qubits (i.e., fixed *d*).

**Fig. 4:**
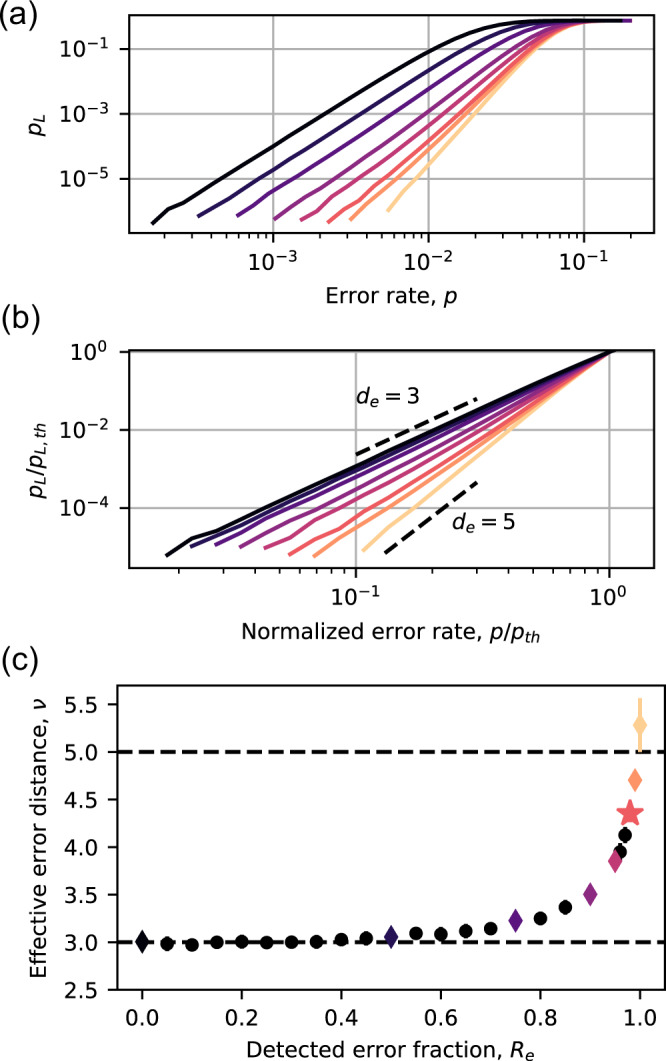
Logical error scaling below threshold. **a** *p*_*L*_ vs *p* at a fixed code distance *d* = 5 for various values of *R*_*e*_ [colors correspond to the diamond points in panel (**c**)]. In panel (**b**), the physical and logical error rates are rescaled by their values at the threshold. **c** Logical error exponent *ν*, extracted from the slope of the curves in (**b**). The dashed lines show the expected asymptotic exponents for pure computational errors (*ν*_*p*_ = 3) and pure erasure errors (*ν*_*e*_ = 5). The error bars indicate the 95% confidence interval in the exponent *ν*, estimated from a chi-squared analysis of the fit.

In Fig. 4a, we plot the logical error rate as a function of the physical error rate for a *d* = 5 code for several values of *R*_*e*_. When normalized by the threshold error rates (Fig. 4b), it is evident that the exponent (slope) *ν* increases with *R*_*e*_. The fitted exponents (Fig. 4c) smoothly increase from the expected value for pure Pauli errors, *ν*_*p*_ = (*d* + 1)/2 = 3, to the expected value for pure erasure errors, *ν*_*e*_ = *d* = 5 (in fact, it exceeds this value slightly in the region sampled, which is close to the threshold). For *R*_*e*_ = 0.98, *ν* = 4.35(2). Achieving this exponent with pure Pauli errors would require *d* = 7, using nearly twice as many qubits as the *d* = 5 code in Fig. 4. For very small *p*, the exponent will eventually return to *ν*_*p*_, as the lowest weight failure (*ν*_*p*_ Pauli errors) will become dominant. The onset of this behavior is barely visible for *d* = 5 in Fig. 3a.

## Discussion

There are several points worth discussing. First, we note that the threshold error rate for *R*_*e*_ = 0.98 corresponds to a two-qubit gate fidelity of 95.9%, which is exceeded by the current state-of-the-art. Recently, entangled states with fidelity ${{{{{{{\mathcal{F}}}}}}}}=97.4\%$ were demonstrated for hyperfine qubits in Rb^35^, and we also note that ${{{{{{{\mathcal{F}}}}}}}}=99.1\%$ has been demonstrated for ground-Rydberg qubits in ^88^Sr^52^. With reasonable technical improvements, a reduction of the error rate by at least one order of magnitude has been projected^37^, which would place neutral atom qubits far below the threshold, into a regime of genuine fault-tolerant operation. Arrays of hundreds of neutral atom qubits have been demonstrated^53,54^, which is a sufficient number to realize a single surface code logical qubit with *d* = 11, or five logical qubits with *d* = 5. While we analyze the surface code in this work because of the availability of simple, accurate decoders, we expect erasure conversion to realize a similar benefit on any code. In combination with the flexible connectivity of neutral atom arrays enabled by dynamic rearrangement^55–57^, this opens the door to implementing a wide range of efficient codes^58^.

Second, in order to compare erasure conversion to previous proposals for achieving fault-tolerant Rydberg gates by repumping leaked Rydberg population in a bias-preserving manner^15^, we have also simulated the XZZX surface code with biased noise and bias-preserving gates. For noise with bias *η* (i.e., if the probability of *X* or *Y* errors is *η* times smaller than *Z* errors), we find a threshold of *p*_th_ = 2.27% for the XZZX surface code when *η* = 100, which increases to *p*_th_ = 3.69% when *η* → *∞*. For comparison, the threshold with erasure conversion is higher than the case of infinite bias if *R*_*e*_ ≥ 0.96, with the additional benefit of not requiring bias-preserving gates.

Third, we consider the role of imperfect erasure detection, or other sources of atom loss. Since two-qubit blockade gates have well-defined behavior with regard to lost atoms (i.e., the lost atoms act as if they are in $\left|0\right\rangle$), these events can be handled fault-tolerantly with no extra ancillas and only one extra gate per stabilizer measurement, using the "quick circuit" for leakage reduction introduced in ref. 17. In that work it was shown that the impact on the threshold was very slight if the loss probability was small compared to other errors^17^, and the same behavior can be expected in the scheme considered here. We leave a detailed analysis to future work.

Fourth, our analysis has focused on two-qubit gate errors, since they are dominant in neutral atom arrays, and are also the most problematic for fault-tolerant error correction^59^. However, with very efficient erasure conversion for two-qubit gate errors, the effect of single-qubit errors, initialization and measurement errors, and atom loss may become more significant. In Supplementary Note 6, we present additional simulations showing that the inclusion of initialization, measurement, and single-qubit gate errors with reasonable values does not significantly affect the threshold two-qubit gate error. We also note that erasure conversion can also be effective for other types of spontaneous errors, including Raman scattering during single qubit gates, the finite lifetime of the ^3^*P*_0_ level, and certain measurement errors.

Lastly, we highlight that erasure conversion can lead to more resource-efficient, fault-tolerant subroutines for universal computation, such as magic-state distillation^60^, which uses several copies of faulty resource states to produce fewer copies with lower error rate. This is expected to consume large portions of the quantum hardware^59,61^, but the overhead can be reduced by improving the fidelity of the input raw magic states. By rejecting resource states with detected erasures, the error rate can be reduced from *O*(*p*)^62–65^ to *O*((1 − *R*_*e*_)*p*). Therefore, 98% erasure conversion can give over an order of magnitude reduction in the infidelity of raw magic states, resulting in a large reduction in overheads for magic state distillation.

While this work provides a novel motivation to pursue qubits based on Yb and other alkaline earth-like atoms, these atoms have also attracted recent interest thanks to other potential advantages^28,29,40,52,66–69^. In particular, long qubit coherence times^28,29,69^, narrow-line laser cooling, and rapid single-photon Rydberg excitation from the metastable ^3^*P*_0_ level offer the potential for improved entangling gate fidelities and a suppression of technical noise. We note that the highest reported Rydberg entanglement fidelity was achieved using the analogous metastable state in ^88^Sr^52^. The use of a metastable electronic level offers other benefits, including straightforward mid-circuit measurement and array reloading capabilities, as demonstrated recently in the context of trapped ions^30,70,71^.

In conclusion, we have proposed an approach for efficiently implementing fault-tolerant quantum logic operations in neutral atom arrays using ^171^Yb. By leveraging the unique level structure of this alkaline earth atom, we convert the dominant source of error for two-qubit gates—spontaneous decay from the Rydberg state—into directly detected erasure errors. We find a 4.4-fold increase in the circuit-level threshold for a surface code, bringing the threshold within the range of current experimental gate fidelities in neutral atom arrays. Combined with a steeper scaling of the logical error rate below the threshold, this approach is promising for demonstrating fault-tolerant logical operations with near-term experimental hardware. We anticipate that erasure conversion will also be applicable to other codes and other physical qubit platforms^30^.

## Methods

### Error correcting code simulations

In this section, we provide additional details about the simulations used to generate the results shown in Figs. 3 and 4. We assign each two-qubit gate to have an error from the set {*I*, *X*, *Y*, *Z*}^⊗2^\{*I* ⊗ *I*} with probability *p*_*p*_/15, and an erasure error with probability *p*_*e*_, with *p*_*e*_/(*p*_*p*_ + *p*_*e*_) = *R*_*e*_. Immediately after an erasure error on a two-qubit gate, both qubits are re-initialized in a completely mixed state which is modeled using an error channel (*IρI* + *XρX* + *YρY* + *ZρZ*)/4 on each qubit. We choose this model for simplicity, but in the experiment, better performance may be realized using an ancilla polarized into $\left|1\right\rangle$, as Rydberg decays only happen from this initial state. In addition, we note that the majority of errors result in only one of the atoms leaving *Q* (Supplementary Note 4), but the other atom has an error anyway and should still be considered as part of the erasure. We assume the existence of native CZ and CNOT gates, so a stabilizer cycle can be completed without single-qubit gates. We also neglect idle errors, since these are typically insignificant for atomic qubits.

Ancilla initialization (measurement) are handled in a similar way, with a Pauli error following (preceding) a perfect operation, with probability *p*_*m*_ (*p*_*m*_ = 0 in Figs. 3 and 4, but results for *p*_*m*_ > 0 are discussed in Supplementary Note 6).

We simulate the surface code with open boundary conditions. Each syndrome extraction round proceeds in six steps: ancilla state preparation, four two-qubit gates applied in the order shown in Fig. 1, and finally a measurement step. For a *d* × *d* lattice, we perform *d* rounds of syndrome measurements, followed by one final round of perfect measurements. The decoder graph is constructed by connecting all space-time points generated by errors in the circuit applied as discussed above. Each of these edges is then weighted by ${{{{\rm{ln }}}}}(p^{\prime} )$ truncated to the nearest integer, where $p^{\prime}$ is the largest single error probability that gives rise to the edge. After sampling an error, the weighted UF decoder is applied to determine error patterns consistent with the syndromes. We do not apply the peeling decoder but account for logical errors by keeping track of the parity of the number of defects crossing the logical boundaries. Our implementation of the decoder was separately benchmarked against the results in ref. 49 and yields same thresholds.

For the comparison to the threshold of the XZZX code when the noise is biased, we apply errors from *Q* = {*I*, *X*, *Y*, *Z*}^⊗2^\{*I* ⊗ *I*} after each two qubit gate with probability *p*_*Q*_. The first (second) operator in the tensor product is applied to the control (target) qubit. In the case of CNOT, we assume bias-preserving gates, and thus set *p*_*ZI*_ = *p*, _*pIZ*_ = *p*_*ZZ*_ = *p*/2 with the probability of other non-pure-dephasing Pauli errors set to *p*/*η*^13^. For the CZ gate we use *p*_*ZI*_ = *p*, _*pIZ*_ = *p* with the probability of other non-pure-dephasing Pauli errors set to *p*/*η*. For the threshold quoted in the main text no single-qubit preparation and measurement noise is applied, to facilitate direct comparison to the threshold with erasure conversion in Fig. 3. In the main text we quote threshold in terms of the total two-qubit gate infidelity ~2*p* for large *η*, to facilitate comparison to the threshold in Fig. 3.

Lastly, we note that the no-jump evolution discussed in Fig. 2b is described by the Kraus operator ${K}_{nj}=I+(\sqrt{(1-p)}-1)\left|1\right\rangle \left\langle 1\right|\approx I-(p/2)\left|1\right\rangle \left\langle 1\right|$ (for small *p*), where *p* is the decay probability. The Pauli-twirl approximation (PTA) reduces any error channel to a Pauli error channel by removing off-diagonal terms in the process matrix. Under the PTA, the non-Hermitian operator *K*_*nj*_ effectively applies a Pauli-Z error at a rate ∝ *p*^2^. This error model is similar to the amplitude damping channel, and previous work has found that the performance of the surface code with the PTA is identical to the exact amplitude damping channel^72^.

## Supplementary information

Supplementary Information

## Data Availability

The Monte Carlo simulation data of the error correcting code performance generated in this study have been deposited in the Harvard Dataverse database under accession code 10.7910/DVN/H9LV4H.
