# Supplementary material for: Erasure conversion for fault-tolerant quantum computing in alkaline earth Rydberg atom arrays
Source: Nat Commun. 2022 Aug 9;13:4657. doi: 10.1038/s41467-022-32094-6 (PMC9363413; doi:10.1038/s41467-022-32094-6)
Supplement: Supplementary file 1 — Supplementary Information [file 41467_2022_32094_MOESM1_ESM.pdf]

Supplementary Information for: "Erasure conversion for fault-tolerant quantum computing in alkaline earth Rydberg atom arrays"

Yue Wu,[1] Shimon Kolkowitz,[2] Shruti Puri,[3] and Jeff D. Thompson[4,*]

[1] *Yale University, Department of Computer Science, New Haven, CT 06520*
[2] *University of Wisconsin-Madison, Department of Physics, Madison, WI 53706*
[3] *Yale University, Department of Applied Physics, New Haven, CT 06520*
[4] *Princeton University, Department of Electrical and Computer Engineering, Princeton, NJ, 08544*
(Dated: July 13, 2022)

Supplementary Note 1. ^{171}YB GATE OPERATIONS

Here, we provide a sketch of a universal set of gate operations on qubits encoded in the metastable $^3\mathrm{P}_0$ level of ^{171}Yb. These closely follow techniques for ground state qubits. Metastable qubit gate operations have also been proposed [1] and demonstrated [2] with trapped atomic ions.

Starting with an atom in $^1\mathrm{S}_0$, initialization into $|1\rangle$ can be performed by optically pumping into $\left|^1\mathrm{S}_0, m_F = 1/2\right\rangle$ and transferring to the $^3\mathrm{P}_0$ (manifold Q) using the clock transition. Mid-circuit measurement can be performed using the same clock pulse to selectively transfer population in $|1\rangle$ to $^1\mathrm{S}_0$, and measuring the $^1\mathrm{S}_0$ population with fluorescence. As an alternative to driving the clock transition, optical pumping via intermediate S and D states can also be used.

Single qubit gate rotations can be performed using Raman transitions and light shifts on the $6s7s$ $^3\mathrm{S}_1$ transition (649 nm), or via the Rydberg state. In both cases, errors can arise from photon scattering, but erasure conversion can be performed at a similar or greater level than for the two-qubit gates discussed in the main text (see section Supplementary Note 5).

Supplementary Note 2. YB BRANCHING RATIOS

In this section, we consider the decay pathways from the Rydberg state, which determine the probability that a spontaneous decay is converted into an erasure. These calculations involve dipole matrix elements between ground states and Rydberg states in Yb that have not been directly measured or computed with rigorous many-body techniques. Therefore, we estimate them using a single active electron approximation [3], and wavefunctions computed using the Numerov technique [4]. We focus on the $6s75s$ $^3\mathrm{S}_1$ $F = 3/2$ state for concreteness [5].

The decay pathways can be separated into BBR decays to nearby n and radiative decays to low-n states. For $n = 75$, the BBR decay rate is 3480 1/s, and the radiative decay rate is 2200 1/s, which gives a branching ratio of 0.39 into radiative decay, and 0.61 into BBR decay (Fig. 1a).

The radiative decays favor the lowest energy states, because of the larger density of states at the relevant transition energy [6]. However, angular momentum algebra favors higher J states within the same fine structure manifold. Therefore, the fraction of decays that terminate directly in the $J = 0$ qubit manifold Q is only 0.025 (Fig. 1b).

Decay events to $6s6p$ $^3\mathrm{P}_1$ will quickly relax to the ground state $^1\mathrm{S}_0$ via a second spontaneous decay. Decays to $6s6p$ $^3\mathrm{P}_2$ can be repumped to $6s6p$ $^3\mathrm{P}_1$ via $6s5d$ $^3\mathrm{D}_2$, which cannot decay to the qubit subspace because of angular momentum selection rules.

However, approximately 0.17 of all the decay events are to $6snp$ states with $n > 6$. These states will overwhelmingly decay to the $6s7s$ $^3\mathrm{S}_1$ and $6s5d$ $^3\mathrm{D}_J$ states, which in turn can decay to $6s6p$ states (Fig. 1c). No data is available to estimate the relative branching ratio between the S and D decay pathways, but we can estimate the fraction of decays that return to Q within each pathway.

The state $6s7s$ $^3\mathrm{S}_1$ decays into the $6s6p$ $^3\mathrm{P}_J$ levels with a branching ratio that can be estimated as [7]:

$$\frac{\Gamma_J}{\Gamma_{tot}} = \frac{1}{\mathcal{N}} \omega_J^3 (2J+1)(2L'+1) \begin{Bmatrix} L & L' & 1 \\ J' & J & S \end{Bmatrix}^2 \tag{1}$$

Here, the primed quantities denote the angular momenta of the initial state ($^3\mathrm{S}_1$), and the unprimed quantitites for the final state ($^3\mathrm{P}_J$). ω_J is the transition frequency for the decay to the state J, and the normalization constant

* jdthompson@princeton.edu

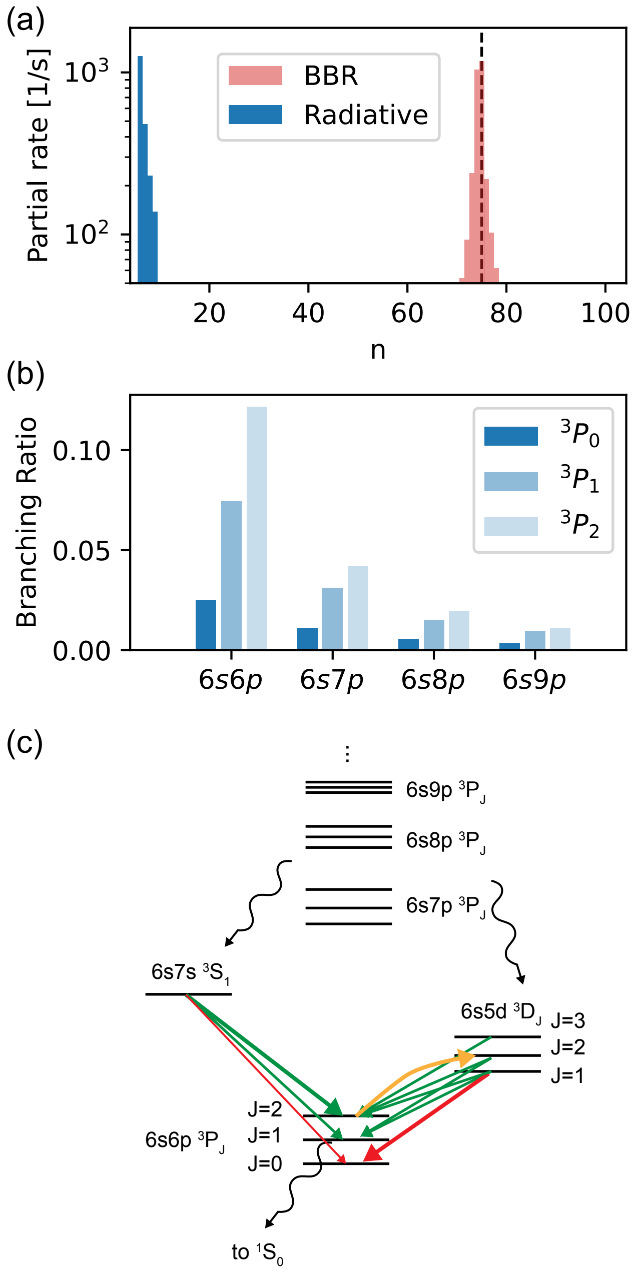

Supplementary Figure 1. Decay pathways from the Yb ^{3}S$_1$ Rydberg series. (a) Partial decay rates to all P states of a given principal quantum number n, starting from $n = 75$. BBR transitions to nearby states are shown in red, and radiative decay to lower n states are shown in blue. Final states with $n = 10 - 35$ are not included because of the absence of spectroscopic data. (b) Branching ratio into low-energy $6sps$ ^{3}P$_J$ states. The branching ratio into the qubit manifold Q, $6s6p$ ^{3}P$_0$, is 2.5%. (c) Partial energy diagram showing relevant transitions between low-lying states. Decays in red are to Q, while the orange transition can be used to repump $^3P_2$ without populating Q.

$\mathcal{N}$ ensures $\sum_J \Gamma_J = \Gamma_{tot}$. The branching ratios into $J = \{0, 1, 2\}$ are $\{0.15, 0.40, 0.45\}$. Therefore, around 0.15 of all decays via ^{3}S$_1$ will reach Q.

In the case of decays via the $6s5d$ ^{3}D$_J$ states, we can use Eq. (1) to estimate the branching ratio from $6snp$ ^{3}P$'_J$ to the various ^{3}D$_J$ states. Since only ^{3}D$_1$ can decay to the Q, we only state this fraction, which is approximately $\{1, 0.25, 0.01\}$ when starting from $6snp$ $^3P_{J'}$ with $J' = \{0, 1, 2\}$. To estimate the branching ratio from $6s5d$ ^{3}D$_1$ to Q, we do not use Eq. (1) because the states are rather close in energy, but instead use the theoretical matrix elements in Ref. [8], which give a branching ratio of 0.65. Combining this with the distribution of population among the $6snp$ $^3P_{J'}$ levels in Fig. 1b, we arrive at an estimate that 0.16 of the decays via D states terminate in Q.

As the probability to end up in Q via the S or D decay pathways is similar, the (unknown) branching ratio between

them becomes unimportant. Taking it to be 0.5, we conclude that 14% of decays from $6snp$ levels with $n > 6$ return to Q. Adding this to the direct decays to Q, we arrive at a final estimate that 0.051 of all Rydberg decays return to the qubit manifold Q.

Lastly, we note that this analysis does not include the effect of doubly-excited states that perturb the Rydberg series, which can give rise to additional decay pathways [9]. In Yb, these are especially prominent because of the number of core excited states [10]. There is not enough spectroscopic data about the Yb Rydberg series to quantitatively evaluate the impact of series perturbers. However, we note that these doubly excited states will require a minimum of three spontaneous decays to reach the $6s6p$ $^3\mathrm{P}_J$ states. Given the general propensity to decay to higher J states at each step, it is likely that the branching ratio into $^3\mathrm{P}_0$ from doubly-excited perturbers will not be worse than the values estimated above.

We also do not explicitly include hyperfine structure in these calculations, but rather calculate matrix elements between J states in ^{174}Yb. This is an excellent approximation for the transitions from low-n to Rydberg states, since these matrix elements are mainly sensitive to the Rydberg state quantum defect, and the $^3\mathrm{S}_1$ $F = 3/2$ Rydberg state that we consider has the same quantum defect as the $^3\mathrm{S}_1$ series in ^{174}Yb [11] because its core electron configuration is purely Yb$^+$ $F = 1$. However, it is possible that the BBR transition rate varies slightly between isotopes, since the hyperfine splitting changes the energy level spacing by a significant amount. We believe that the error from this approximation is much less than the uncertainty arising from unknown series perturbers.

Supplementary Note 3. ERASURE DETECTION FIDELITY

A. Detection of atoms in $^1S_0$

We first consider the localized detection fidelity for atoms $^1S_0$, using the cycling transition in the R manifold. Many protocols for imaging atoms in tweezers focus on non-destructive detection, and therefore image slowly while simultaneously cooling, which is not optimal for minimizing computational cycle time [12]. Here we instead consider rapid but destructive detection [13], with the aim of replacing atoms from a reservoir when erasures are detected (which occurs with a low probability). To estimate the fidelity, we take the atoms to be initially at rest, ignore the dipole trap, and assume illumination by counter-propagating fields above saturation, such that the photon scattering rate is $\Gamma/2$. This results in no net force on the atom, but momentum diffusion from photon recoils leads to an increasing mean squared atomic displacement of [13, 14]:

$$\langle x^2(t)\rangle = \frac{v_{rec}^2}{3}\frac{t^3}{3}\frac{\Gamma}{2} = \frac{\hbar^2 k^2}{18m^2}t^3\Gamma \tag{2}$$

where $\Gamma = 2\pi \times 28$ MHz is the $^1S_0$ - $^1P_1$ transition linewidth, the wavevector $k = 2\pi/\lambda$ with $\lambda = 399$ nm, and m is the atomic mass.

We envision a tweezer array with a spacing of $a = 3 - 5\,\mu$m, and therefore require that $\sqrt{\langle x^2(t)\rangle} < a/2$ to determine which site is fluorescing. In Ref. [13], free-space imaging of single ^{6}Li atoms was demonstrated with a detection fidelity of 99.4% after an imaging time of 20 µs, after which time $\sqrt{\langle x^2(t)\rangle}^{1/2} = 10.4$ µm. During this time, approximately 330 photons were scattered, and 25 detected, with an EMCCD and a modest numerical aperture objective (NA=0.55). However, for the same number of detected photons, the position spread scales as $1/(m\lambda\Gamma)$ [13], and this quantity is a factor of 81 smaller for the heavy ^{171}Yb compared to ^{6}Li, so we anticipate a position spread of only 120 nm for the same conditions. Therefore, achieving imaging fidelity greater than 99.9% should be readily achievable for atoms in $^1S_0$, in less than half the time, since the scattering rate for Yb is more than 3 times larger.

B. Detection of ions

We now consider the detection fidelity of Yb$^+$ ions using the cycling transition in manifold B following autoionization out of a Rydberg state. Ions created from Rydberg atoms have been imaged using fluorescence in ultracold quantum gases of strontium [15], although we note that only ensembles with $N \approx 10^5$ ions were studied in that work, and not single ions. Compared to detecting neutral atoms, there are two additional factors that make ion detection more challenging: an initial velocity v_0 arising from recoil momentum from the ejected electron, and acceleration due to a background electric field or the presence of other ions. We begin by considering the initial velocity: when a $6p_{1/2}np$ Rydberg state decays to Yb$^+$ $(6s)$ + e^- via autoionization, the electron carries away an energy $\Delta E \approx I_{6p_{1/2}} - I_{6s} \approx 27100\,\mathrm{cm}^{-1}$, where I_j is the ionization limit for Yb^0 corresponding to the ion core in state j,

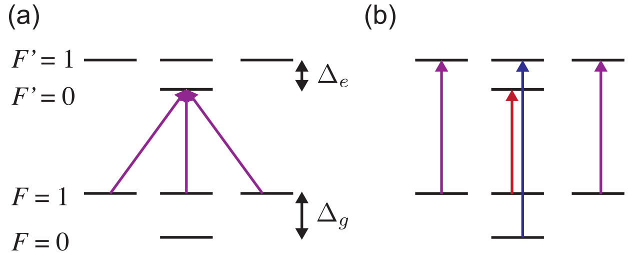

Supplementary Figure 2. (a) Typical driving approach for hyperfine state readout on the ^{171}Yb$^+$ $6s_{1/2} \to 6p_{1/2}$ transition [17]. The $F \to F-1$ transition has two dark states (remixed by a magnetic field, not shown) that reduces the fluorescence by a factor of 1/3, to a maximum of $\Gamma/6$. (b) Three-frequency driving scheme to avoid dark states, achieving a saturated photon scattering rate of $\Gamma/2$.

and we have made the approximation that the electron mass is very small compared to the ion mass. In this case, the ion acquires a recoil momentum $p_e = \sqrt{2\Delta E m_e}$, corresponding to a velocity $v_0 = p_e/m \approx 3.5$ m/s.

With a finite initial velocity, the mean squared position is:

$$\langle x^2(t)\rangle = v_0^2 t^2 + \frac{\hbar^2 k^2}{18m^2} t^3 \Gamma = v_{rec}^2 t^2 \left[\left(\frac{v_0}{v_{rec}}\right)^2 + \frac{t\Gamma}{18}\right] \tag{3}$$

where $v_{rec} = \hbar k/m$ is the recoil velocity for the imaging wavelength, now 369 nm, and $\Gamma = 2\pi \times 19$ MHz. For the parameters above, $v_0/v_{rec} \approx 550$. Recognizing that the number of scattered photons is $N_{ph} = t\Gamma/2$, it is clear that the first term dominates for $N_{ph} < 10^6$. Therefore, we can express the position as:

$$\sqrt{\langle x^2(t)\rangle} = \frac{2 v_0 N_{ph}}{\Gamma} \approx 54\,\text{nm/photon} \tag{4}$$

With a total detection efficiency of $\eta = 0.1$, an average of 5 photons can be detected while maintaining $\sqrt{\langle x^2(t)\rangle} < 2.5\,\mu$m, corresponding to 99% detection fidelity in the absence of dark counts. The necessary imaging time is less than 2 μs.

Achieving this collection and detection efficiency is challenging but achievable. A lens with NA=0.7 collects approximately 14% of the light from an unpolarized emitter (double-sided NA=0.7 lenses have recently been demonstrated in a tweezer setup [16], giving twice the efficiency), and EMCCD detectors with a quantum efficiency of 75% are also commercially available (*i.e.*, NuVu Cameras HNu512 with blue-enhanced sensor). We also note that fluorescence detection of the hyperfine state in ^{171}Yb$^+$ typically results in a saturated fluorescence rate 3-4 times slower than $\Gamma/2$ [17]. This arises from coherent dark states on the $F = 1 \to F' = 0$ transition used for state readout (Fig. 2a). However, we only wish to detect the presence of the ion and not its state. Driving both hyperfine levels simultaneously with multiple frequency tones (Fig. 2b) can eliminate these dark states, resulting in a saturated fluorescence rate of $\Gamma/2$.

We note that this is a conservative estimate for several reasons. First, Yb$^+$ ions are only produced on atoms undergoing a two-qubit gate, and these gates cannot be performed on every atom in the array in parallel because of cross-blockade effects. Therefore, it is only necessary to resolve the atoms participating in gates in a particular cycle, which may have a separation of $2a$ or $3a$, allowing for longer imaging times and more particle spread. Second, we have assumed that the recoil momentum is always in the plane of the array. However, out-of-plane motion does not affect the in-plane imaging resolution, and the true momentum distribution may be isotropic or even preferentially out-of-plane depending on the polarization of the autoionization light [18]. Lastly, we have treated all autoionization events as transitions to Yb$^+$ ($6s$), while in reality, a significant fraction of autoionization events will decay to a Yb$^+$ ($5d$) state. These states can be quickly repumped to 6s, so imaging can proceed as normal. However, for this decay process, ΔE is smaller by a factor of approximately 6, and v_0 is smaller by a factor of 2.6, significantly relaxing the requirements on the imaging parameters for these atoms. To the best of our knowledge, the branching ratio to the $5d$ states has not been measured for Yb $6p_{1/2}ns$ states, though the analogous quantity has been studied in Ba [19] and can be between 15-88%, depending on the initial state.

We can also consider the role of a background electric field, which will cause a position displacement:

$$\Delta x = \frac{qE}{2m} t^2 = \frac{qE}{2m}\left(\frac{2N_{ph}}{\Gamma}\right)^2 \tag{5}$$

Here, E is the field strength and q is the electron charge. Using the Yb^+ ion parameters and $N_{ph} = 200$, this results in a drift of approximately 316 nm/(mV/cm) during the imaging time. With intra-vacuum electrodes, it is possible to null background electric fields at the level of approximately 1 mV/cm [5], so this is not a significant source of imaging error.

Lastly, we consider electric fields resulting from the simultaneous creation of multiple Yb^+ ions in a single gate cycle. We are not concerned with ions created in previous gate cycles, because they can be cleared with a brief electric field pulse. In the main text, and in section Supplementary Note 4, we predict that the probability of generating two ions in a single two-qubit gate is extremely small due to the Rydberg blockade. Therefore, in this section we are concerned with the probability to create ions in nearby gates. First, let us estimate the tolerable separation between ions. The electric field produced by a single ion is 16 mV/cm at a distance $d = 30\,\mu$m. If two ions are created at this separation, the resulting force will cause a displacement of approximately 5 μm during the time it takes to scatter 200 photons, which is roughly comparable to the targeted spatial resolution. Therefore, we conclude that $d_i = 30\,\mu$m is the minimum ion separation that can be tolerated without incurring additional detection errors. If gates are performed across the array with a separation of d_g, the probability of a multi-ion event impairing the detection of a given ion is $(N_g - 1)p$, where $N_g \approx (d_i/d_g)^2$ is the number of parallel gates within d_i. This should be compared to the intrinsic ion detection infidelity to estimate whether multi-ion events will degrade the average detection fidelity. For example, given a detection fidelity of 99% for isolated ions, and a gate error of $p = 10^{-3}$ (see section Supplementary Note 4), any value of $d_g > 10\,\mu$m will not significantly alter the overall ion detection probability. This is compatible with performing parallel gates every three sites in a 2D array with $a = 3\,\mu$m. The impact of multi-ion events on detection fidelity can be reduced further at the expense of gate parallelism and operation time (see section Supplementary Note 7).

C. Alternate detection strategies for population in Rydberg states

Fluorescence detection of ions has the benefit of being fast and compatible with existing experimental techniques. One alternate approach is to detect Yb^+ ions and electrons using charged particle optics and detectors. A second alternative is to simply wait for any Rydberg atoms to decay. To ensure more than 99.9% of the ions have decayed, it would be necessary to wait approximately $\tau > 7/\Gamma \approx 1$ ms, and avoiding atom loss during this time will require that all of the intermediate Rydberg states are trapped. However, this is straightforward in alkaline earth atoms using the polarizability of the ion core [5]. Because of the large number of intermediate Rydberg states and their complex radiative decay pathways, it is not possible to accurately calculate the ultimate branching ratio back into $^3P_0$, but a crude estimate suggests it would result in less efficient erasure conversion, with $R_e \approx 0.9$.

Supplementary Note 4. GATE SIMULATIONS

In this section, we describe a detailed, microscopic simulation of a two-qubit gate using the level structure in Fig. 1, to evaluate the quantitative performance of the erasure conversion approach. While we expect that this protocol should work for any Rydberg gate, we focus specifically on the protocol introduced in Ref. [20], and applied to ^{171}Yb in Ref. [11], which we refer to hereafter as the *LP gate*.

The system is described by the following two-atom Hamiltonian:

$$\begin{aligned} H = \sum_{i=\{1,2\}} & \frac{1}{2}\left(\Omega \left|r\right\rangle_{ii} \left\langle 1\right| + \Omega^* \left|1\right\rangle_{ii} \left\langle r\right|\right) + \Delta \left|r\right\rangle_{ii} \left\langle r\right| \\ & + V_{rr} \left|rr\right\rangle \left\langle rr\right| + V_{pp} \left|pp\right\rangle \left\langle pp\right| + V_{rp}(\left|rp\right\rangle \left\langle pr\right| + h.c.) \end{aligned} \tag{6}$$

The qubit state $|1\rangle$ in each atom is coupled to $|r\rangle$ by a drive Ω with detuning Δ. The Rydberg blockade shifts the state $|rr\rangle$ by V_{rr}. We also incorporate a single additional state, $|p\rangle$, that is populated by BBR transitions. This state has a a self-blockade interaction with strength V_{pp}, and a cross-blockade interaction with $|r\rangle$ with strength V_{rp}. Only states with large matrix elements to $|r\rangle$ are populated by BBR transitions, and therefore, V_{rp} is dominated by the strong dipole-dipole interaction. Therefore, we expect that $V_{rp} \gg V_{pp}, V_{rr}$.

The LP gate protocol is based on the fact that, when $V_{rr} \gg \Omega$, the initial state $|11\rangle$ cannot be excited to $|rr\rangle$, but is instead excited to $|W\rangle = (|1r\rangle + |r1\rangle)/\sqrt{2}$ at a rate $\sqrt{2}\Omega$. Therefore, the use of an appropriate detuned pulse with a phase slip allows for excitation trajectories for all initial states that return to themselves, but with different accumulated phases for $|11\rangle$ and $|01\rangle$ (or $|10\rangle$), giving rise to a controlled-Z (CZ) gate (Fig. 3a,b) [20].

As discussed in the main text, the dominant, fundamental source of error is decay from $|r\rangle$ during the gate. This can result in a BBR transition to another Rydberg state, a radiative decay to the ground state $^1S_0$ ($|g\rangle$)

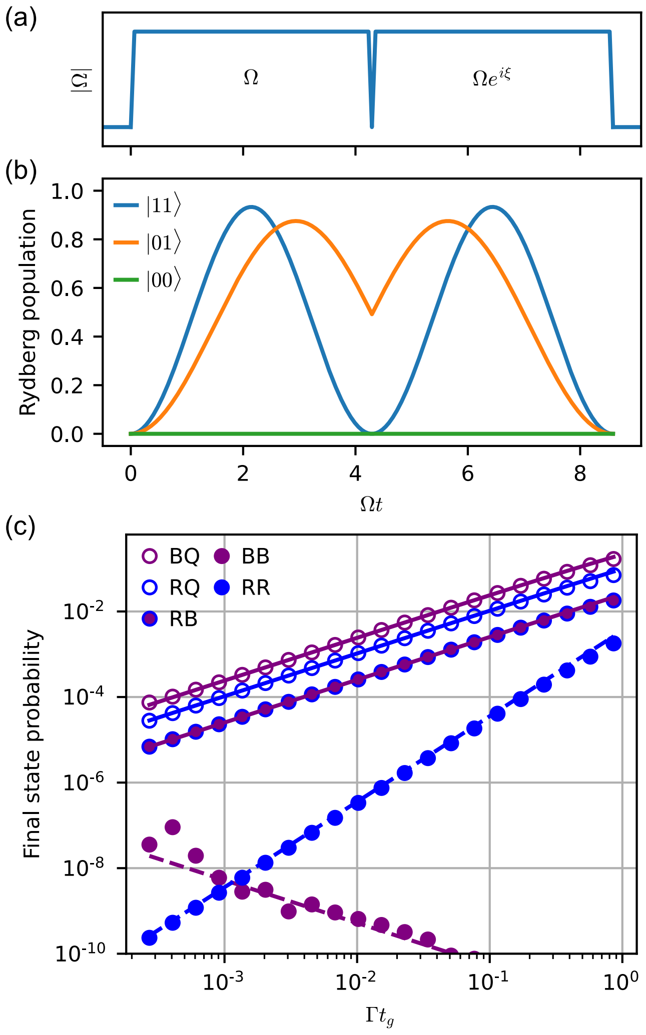

Supplementary Figure 3. Gate simulations. (a) Pulse sequence used to implement the CZ gate from Ref. [20]. (b) Rydberg state population during the gate, for various initial states. (c) Probability of individual erasure error channels (see Fig. 2a). The solid lines are analytic estimates from section Supplementary Note 4.

or the computational level. During the erasure detection step, these correspond to three distinct outcomes: ion fluorescence (which we abbreviate B), ground state fluorescence (R) or no signal, indicating that the qubit remains in the computational space (Q). In a two-qubit gate, the outcome QQ signals no erasure, while any other outcome is considered to be an erasure error on both qubits.

A. Analytic error model

In this section, we derive analytic expressions for the probabilities of various errors to occur during the two-qubit gate. For atoms beginning in the state $|00\rangle$, there is no excitation to the Rydberg state, and therefore no errors. Below, we consider the other initial states.

1. *Initial state* $|01\rangle$ *(or* $|10\rangle$*)*

First, consider the case that the atoms start in $|01\rangle$. The case $|10\rangle$ is identical because the gate is symmetric in the two atoms. During the gate, in the absence of errors, we can represent the state of the atoms as:

$$|\psi(t)\rangle = \psi_1(t)\,|01\rangle + \psi_r(t)\,|0r\rangle \tag{7}$$

The Rydberg excitation probability $|\psi_r(t)|^2$ is plotted in Fig. 3b.

The probability of a blackbody decay that leaves the qubits in the configuration QB (Fig. 4a) is given by the decay rate Γ_B and the average population in the Rydberg state during the gate, α:

$$\Gamma_B \alpha t_g = \Gamma_B \int_0^{t_g} |\psi_r(t)|^2 \, dt \tag{8}$$

Similarly, the probability of a radiative decay to QR is $\Gamma_R \alpha t_g$. For the LP gate, $\alpha \approx 0.532$.

The probability of the qubit decaying back to the computational space is $\Gamma_Q \alpha t_g$. We make two simplifying assumptions about this process. First, we set the decay probability to $|00\rangle$ and $|01\rangle$ to be equal, though in reality they are biased towards $|01\rangle$, which is more favorable. Second, we assume that the time spent in intermediate states is negligible compared to t_g, which is well-justified if $t_g > 100$ ns. After decaying to $|00\rangle$, the qubits will remain there for the rest of the gate. Decays to $|01\rangle$, however, result in re-excitation, resulting in $|0r\rangle$ population at the end of the gate, which is detected as a QB configuration. We denote the fraction of decays to $|01\rangle$ that are re-excited as R_{01}, which we compute as a weighted average over the possible decay times:

$$R_{01} = \frac{1}{t_g \alpha} \int_0^{t_g} |\psi_r(t)|^2 \, |\psi_r(t_g - t)|^2 \, dt \approx 0.700 \tag{9}$$

Here, $|\psi_r(t_g - t)|^2$ is the probability for an atom that has decayed at a time t to be found in $|r\rangle$ at the end of the gate. To see why this is the case, consider the directly computed re-excitation probability: $|\langle r|U(t, t_g)|1\rangle|^2$, where $U(t, t_g)$ is the propagator from time t to t_g. Taking the complex conjugate inside the square modulus allows this to be rewritten as $|\langle r|U(t_g, t)|1\rangle|^2$, describing the evolution of $|1\rangle$ *backwards* in time, from t_g to t. Because the square modulus of the wavefunctions are clearly symmetric around the middle of the gate (Fig. 3b), this can be replaced by $|\langle r|U(0, t_g - t)|1\rangle|^2 = |\psi_r(t_g - t)|^2$.

We can combine these results to arrive at the probability to end up in each subspace, having started in $|01\rangle$:

$$P(QR|01) = \Gamma_R \alpha t_g \tag{10}$$
$$P(QB|01) = \Gamma_B \alpha t_g + (\Gamma_Q/2) \alpha t_g R_{01} \tag{11}$$
$$P(QQ|01) = 1 - P(QR|01) - P(QB|01) \tag{12}$$

2. *Initial state* $|11\rangle$

Now we consider the case that the qubits start in $|11\rangle$ (Fig. 4b). During the gate, with no errors, the state can be represented as:

$$|\psi(t)\rangle = \psi_{11}(t)\,|11\rangle + \psi_W(t)\,|W\rangle + \psi_{rr}(t)\,|rr\rangle \tag{13}$$

where $|W\rangle = (|1r\rangle + |r1\rangle)/\sqrt{2}$. We assume $|\psi_{rr}(t)|^2 \ll 1$ because of the Rydberg blockade, and neglect this component unless otherwise stated. The Rydberg excitation probability $|\psi_W(t)|^2$ is plotted in Fig. 3b.

Proceeding as before, the probability of a blackbody decay to the subspace $QB \cup BQ$ depends on the average Rydberg population β:

$$\Gamma_B \beta t_g = \Gamma_B \int_0^{t_g} |\psi_W(t)|^2 \, dt \tag{14}$$

Similarly, the probability of a radiative decay to $QR \cup RQ$ is $\Gamma_R \beta t_g$. For the LP gate, $\beta \approx 0.467$.

The qubits can also decay to back to the computational space QQ, with a total probability $\Gamma_Q \beta t_g$, and we assume that decays to $|01\rangle$ and $|11\rangle$ happen instantly with equal probability, as discussed in the preceding section. If the decay is to $|11\rangle$, then re-excitation can result in the configuration $QB \cup BQ$ at the end of the gate, with probability R'_{11}:

$$R'_{11} = \frac{1}{t_g \beta} \int_0^{t_g} |\psi_W(t)|^2 \, |\psi_W(t_g - t)|^2 \, dt \approx 0.700 \tag{15}$$

If the decay is to $|01\rangle$, then re-excitation is also possible but with a different probability R'_{11}, given by the single-atom excitation trajectory ψ_r:

$$R_{11} = \frac{1}{t_g \beta} \int_0^{t_g} |\psi_W(t)|^2 \, |\psi_r(t_g - t)|^2 \, dt \approx 0.640 \tag{16}$$

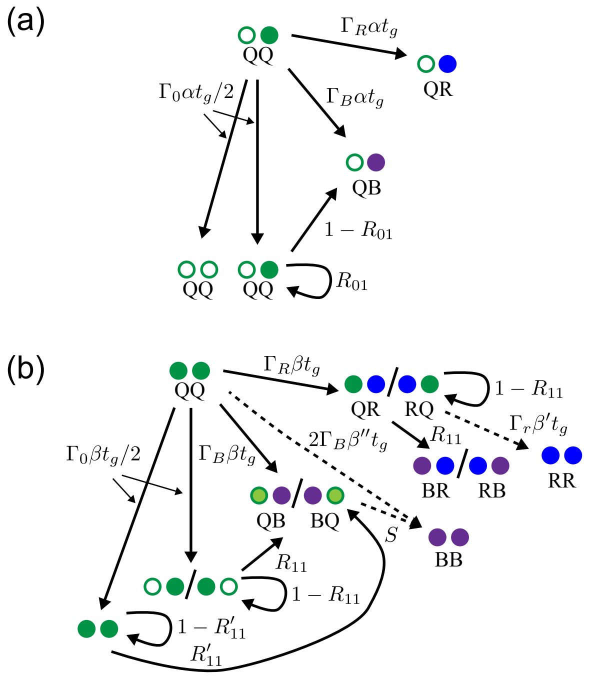

Supplementary Figure 4. Diagram of transition probabilities during a two-qubit gate, for atoms beginning in (a) $|10\rangle$ or (b) $|11\rangle$. See section Supplementary Note 4 A for variable definitions, and Table I for numeric values.

It is also possible that both atoms leave Q, resulting in the configurations $BR\cup RB$, RR or BB. The configuration $BR\cup RB$ can be populated by an initial radiative decay to $QR\cup RQ$, followed by re-excitation of the qubit remaining in Q (which is always in $|1\rangle$). The probability for this to occur is also R_{11}.

The configuration RR can be populated by a second radiative decay after an initial decay to $QR\cup RQ$. The probability for this to occur, conditioned on the first radiative decay, is given by $\Gamma_R\beta' t_g$, where β' is the average Rydberg population after the first decay:

$$\begin{aligned}\beta' &= \frac{1}{t_g\beta}\int_0^{t_g} dt\,|\psi_W(t)|^2\,\frac{1}{t_g}\int_t^{t_g} dt'\,|\psi_r(t_g-t)|^2 \\ &\approx 0.266\end{aligned} \tag{17}$$

Lastly, the configuration BB can be populated in two ways: by re-excitation after an initial blackbody decay to $QB\cup BQ$, or decay from the doubly excited state $|rr\rangle$. The former is strongly suppressed by the blockade term V_{rp} (in Eq. 6), while the latter is strongly suppressed by the blockade V_{rr}. The direct decay from $|rr\rangle$ occurs with probability:

$$2\Gamma_B\beta'' t_g = 2\Gamma_B\int_0^{t_g}|\psi_{rr}(t)|^2\,dt \tag{18}$$

with the average $|rr\rangle$ population $\beta''\approx\beta\Omega^2/(2V_{rr}^2)$. Note that only a single decay is required, as the state $|rp\rangle$ results in the creation of two ions.

The probability for a pair of atoms that has already decayed to $QB\cup BQ$ to be re-excited is:

$$S = \frac{1}{t_g\beta}\int_0^{t_g}|\psi_W(t)|^2\,|\psi_{rp}(t_g-t)|^2\,dt \tag{19}$$

Here, $|\psi_{rp}(t)|^2\approx\Omega^2/(2V_{rp}^2)$ is the probability for the state $|1p\rangle$ to evolve into $|rp\rangle$ after a time t.

From these expressions, we can compute the probability to end up in different final states, starting in $|11\rangle$.

Term	Value
α	0.532
R_{01}	0.700
β	0.467
R_{11}	0.640
R'_{11}	0.700
β'	0.266
β''	$\beta\Omega^2/(2V_{rr}^2)$
S	$\Omega^2/(2V_{rp}^2)$

TABLE I. Coefficients of the transition rates in Fig. 4, evaluated for the CZ gate from Ref. [20].

$$P(QR \cup RQ|11) = \Gamma_R \beta t_g (1 - R_{11}) \tag{20}$$

$$\begin{aligned} P(QB \cup BQ|11) &= \Gamma_B \beta t_g (1 - S) \\ &+ \Gamma_Q \beta t_g (R_{11} + R'_{11})/2 \end{aligned} \tag{21}$$

$$P(RB \cup BR|11) = \Gamma_R \beta t_g R_{11} \tag{22}$$

$$P(RR|11) = (\Gamma_R t_g)^2 \beta\beta' \tag{23}$$

$$P(BB|11) = \Gamma_B \beta t_g S + 2\Gamma_B \beta'' t_g \tag{24}$$

3. Summary

We can combine the analytic estimates above in Eqs. (10)-(12) and Eqs. (20)-(24) to obtain a total probability of each error channel. Given an initial state with probability $\{P_{00}, P_{01}, P_{11}\}$ to be in $\{|00\rangle, |01\rangle \, or \, |10\rangle, |11\rangle\}$, the probability of each error channel is:

$$P_{QR} = P_{01}\Gamma_R \alpha t_g + P_{11}\Gamma_R \beta t_g (1 - R_{11}) \tag{25}$$

$$\begin{aligned} P_{QB} &= P_{01}\Gamma_B \alpha t_g \\ &+ P_{11} \left[\Gamma_B \beta t_g (1 - S) + \Gamma_Q \beta t_g (R_{11} + R'_{11})/2\right] \end{aligned} \tag{26}$$

$$P_{RB} = P_{11}\Gamma_R \beta t_g R_{11} \tag{27}$$

$$P_{RR} = P_{11}(\Gamma_R t_g)^2 \beta\beta' \tag{28}$$

$$P_{BB} = P_{11} \left[2\Gamma_B \beta'' t_g + \Gamma_B \beta t_g S\right] \tag{29}$$

The total erasure probability p_e is given by the sum of the first five terms. The probability of an undetectable leakage error is $p_f = P_{BB}$.

The first three errors scale as t_g; correspondingly, the probability of these events goes as Γt_g, and are the dominant error mechanism for the gate. The fourth expression, P_{RR}, decreases as $(\Gamma t_g)^2$. The final error probability P_{BB}, scales as $\Gamma t_g \Omega^2/(2V^2) \approx \Gamma/(t_g V^2)$ (here, V is the smaller of V_{rr}, V_{rp}, which is typically V_{rr}. While this error probability decreases with Γ, it increases as t_g decreases, as the larger Ω begins to overpower the blockade. As noted in the main text, the error BB is special because it cannot be readily detected and results in atom loss. However, excitation of $|rr\rangle$ causes other, coherent errors in the gate as well. Therefore, maintaining high fidelity gate operation even in the absence of spontaneous decay requires $\Omega/V > 20$ [20]. Since $P_{BB}/P_{QB} \approx \Omega^2/(2V^2)$, it seems that the probability of BB events will generally be smaller than the probability of undetected QB events, given the detection fidelity discussed in section Supplementary Note 3.

A final source of error is the non-Hermitian no-jump evolution that arises under the monitoring realized by the erasure detection [21]. Since erasure errors do not occur from the state $|00\rangle$, and are approximately equally likely from the remaining computational states, the absence of an erasure detection reveals that the atoms are more likely to be in $|00\rangle$, and therefore the renormalized wavefunction contains more amplitude in this state [22]. This can be understood as a rotation of the two-qubit state towards $|00\rangle$ by an angle proportional to p_e, which contributes to the average gate infidelity at the level of approximately $(p_e/4)^2$. This is not a significant contribution to the total error when $p_e \ll 16(1 - R_e) \approx 0.32$.

B. Comparison to numerical simulations

For comparison, we also perform a master equation simulation of the full two-atom model. We consider the error probabilities as a function of the gate duration, t_g, which depends on the Rabi frequency as $t_g \approx 8.586/\Omega$. The gate error depends primarily on the dimensionless quantity Γt_g, but is also sensitive to the blockade strength (in the high-fidelity regime), which we express in dimensionless units as V_{rr}/Γ. For simplicity, we set $V_{rp} = V_{rr}$, though in reality, V_{rp} is larger because it is a first-order process.

For the $n = 75$ $^3S_1$ state in ^{171}Yb, we assume a Rydberg lifetime $\tau = 1/\Gamma = 100$ μs, and $V = 2\pi \times 1.3$ GHz, based on previous measurements in ^{174}Yb [5, 23], giving $V/\Gamma = 10^6$. The achievable value of t_g depends on the details of the experimental setup and excitation laser. However, we note that $\Omega = 2\pi \times 5.5$ MHz has been demonstrated for this state (starting from $^3P_1$) with very modest laser power [23], which would yield $t_g \approx 250$ ns and $\Gamma t_g \approx 2 \times 10^{-3}$.

In Fig. 3c, the predictions of Eqs. (25)-(29) are shown along with a master equation simulation of the two-atom model. The numerical simulation and the analytic model are in excellent agreement.

C. Other factors limiting gate fidelity

In additional simulations, we have also considered a more realistic six-level model including the other m_F sublevels of the $^3S_1$ $F = 3/2$ state, imperfect laser polarization, and the role of finite blockade strength. A large magnetic field serves to detune the transition from $|0\rangle$ to the $m_F = 1/2$ Rydberg state by $\Delta_z = g_f \mu_B B \gg \Omega$. However, a small light shift remains that contributes both a single-qubit and a two-qubit phase, but which can be corrected by adjusting the gate parameters Δ, ξ. Imperfect polarization also generates a small light shift that can be similarly accommodated. The finite blockade modifies the qubit trajectories but can also be incorporated as a correction, as discussed in Ref. [20].

After optimization of the gate parameters, we find that a fidelity with the target CZ gate of $1 - 10^{-5}$ can be realized (in the absence of spontaneous emission) if $\Delta_z/\Omega > 30$ and $V/\Omega > 100$. Using the experimentally demonstrated $\Omega = 2\pi \times 5.5$ MHz and $\Gamma^{-1} = 100\,\mu$s, the average gate fidelity (including spontaneous emission) is $\mathcal{F} \approx 0.999$ (Fig. 2b). Therefore, with $B \gtrsim 90$ G and $V_{rr} \gtrsim 2\pi \times 550$ MHz (corresponding to a tweezer spacing $a < 4.5\,\mu$m), the additional coherent errors can be suppressed below 10^{-5}, which will not impact the conditional fidelity $\mathcal{F}_{\bar{e}}$ in Fig. 2b. These parameter values are based on the measured g-factor and C_6 coefficient for the $^3S_1$ $F = 3/2$ Rydberg states: $g_F = 1.9$ MHz/G and measured $C_6 = 5(3)$ THz μm^6, respectively [11]. This gate performance matches or exceeds recent theoretically predicted gate fidelities in alkali atom qubits [24, 25].

Supplementary Note 5. ERASURE CONVERSION FOR OTHER ERRORS

While we have so far focused on two-qubit gate errors, as they are dominant and most problematic, the metastable state qubit encoding in ^{171}Yb should also allow erasure conversion for other errors. In this section, we briefly sketch these ideas, leaving a detailed analysis for future work.

First, any spontaneous decay or photon scattering occurring on idle qubits in the ^{3}P$_0$ level is an erasure error with very high probability. Spontaneous decay to ^{1}S$_0$ is always detectable. Raman and Rayleigh scattering from the optical tweezer have a vanishing probability of creating errors in the qubit subspace as long as the tweezer detuning is large compared to the hyperfine splitting in other excited states [26]. It can shorten the lifetime of the qubit level by Raman scattering to other ^{3}P$_J$ states, but these decay or are repumped to ^{1}S$_0$, and detected as erasures.

The same logic can be applied to single-qubit gates performed using Raman transitions via the $6s7s$ ^{3}S$_1$ state, as long as the detuning is large compared to the hyperfine splitting in that state [27]. If single-qubit gates are performed through the Rydberg state, then the analysis is the same as that of the two-qubit gate.

Lastly, we note that a significant source of error in current neutral atom gates is technical noise, either from Doppler shifts or frequency and intensity fluctuations of the driving laser. While this source of error is not fundamental, it is a significant practical nuisance. Noise that is slow compared to the duration of a gate, which is often the case for Doppler shifts and intensity noise, can be cancelled using composite pulse sequences [28] or other robust control techniques [29]. Unfortunately, this typically results in a longer total gate duration, increasing the Rydberg decay probability. However, this trade-off may be more advantageous with erasure conversion.

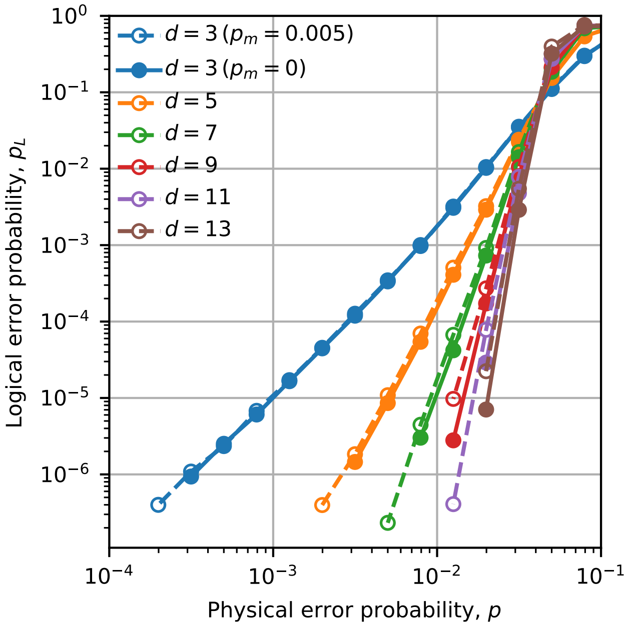

Supplementary Figure 5. Logical error performance with errors on ancilla qubit initialization and measurement with probability $p_m = 0$ (solid lines, filled circles) and $p_m = 0.005$ (dashed lines, open circles).

Supplementary Note 6. IMPACT OF ERRORS IN INITIALIZATION, MEASUREMENT AND SINGLE-QUBIT GATES

In the simulations in Figs, 3 and 4, we assume native operations to initialize and measure the ancillas in the Hadamard basis, and native CNOT and CZ gates, such that no single-qubit gates are required. The impact of single-qubit gate errors can be estimated by considering an alternative stabilizer measurement circuit with ancilla initialization and measurement in the Z basis, and only CZ gates. This requires the insertion of four H gates, which can each be associated with one of the four two-qubit gates. Therefore, a pessimistic assumption is to treat an error in the H as an error in the two-qubit gate, which would increase the two-qubit gate error probabilities to $p_p + p_p^{(1)}$ and $p_e + p_e^{(1)}$, where $p_e^{(1)}/(p_p^{(1)} + p_e^{(1)}) = R_e^{(1)}$ is the erasure fraction of the single qubit gate. If $R_e^{(1)} = 0$, then R_e is reduced by a factor $1/(1 + p^{(1)}/p)$, which means that R_e is not significantly affected if $p^{(1)}/p < 1 - R_e$. This is not an unreasonable assumption for $R_e = 0.98$. However, as discussed above, it is also possible to extend erasure detection to single-qubit gates, which would further relax this requirement.

Additionally, we consider the role of imperfect ancilla initialization and measurement. In the simulation, this is represented by inserting Pauli errors before or after perfect operations with probability p_m. Note that $p_m = 0$ in Figs. 3, 4. Here we attempt to quantify the impact of realistic initialization and measurement errors in two ways. First, we consider a fixed value of p_m. For $p_m = 0.001$, the threshold two-qubit gate error for $R_e = 0.98$ is indistinguishable from its value when $p_m = 0$. If $p_m = 0.005$, we find that the threshold is slightly reduced to $p_{th} = 3.80(2)\%$, but the general behavior, even far below the threshold, is unchanged (Fig. 5). Second, we study the case that the initialization and measurement errors have the same probability as two-qubit gate errors, $p_m = p$. In this case, we find the threshold decreases to $2.85(1)\%$.

Supplementary Note 7. IMPACT OF ERASURE CONVERSION ON OPERATION SPEED

In this section we consider how the operations required for erasure conversion may affect the overall computation speed of a neutral atom quantum computer. A single round of stabilizer measurements for a surface code with distance d requires of order $N = 4d^2$ two-qubit gates, each of which takes a duration $t_g < 1\,us$. Gates that are sufficiently remote can be implemented in parallel [20, 30], and we estimate that in the limit of a large array, a fraction $f_p = 1/10$ of the gates can be applied in each cycle. Therefore, the total time required to apply the gates is $t_g/f_p \approx 10\,\mu$s.

The erasure detection step must occur after each set of parallel gates, and takes a time $t_e \approx 10\,\mu$s (as discussed in

section Supplementary Note 3). This increases the cycle time to $(t_g + t_e)/f_p \approx 100\,\mu$s.

Atom replacement can be deferred until after the stabilizer measurement: once an erasure error has occurred, subsequent gates involving the affected atoms can simply be skipped. The time to move tweezers is t_r, which is several hundred microseconds in recent experiments, [25]. All necessary replacements can be performed in parallel.

Lastly, the ancilla qubits need to be measured to extract the syndrome values, and we denote the time for this operation as t_m. To enable the atoms to be re-used, this measurement should not result in the loss of atoms, which limits the scattering rate and results in $t_m \gtrsim 20$ ms [11, 12].

Therefore, the total duration of a cycle is $(t_g + t_e)/f_p + t_r + t_m$. This is dominated by t_m, and therefore, the erasure conversion protocol will not significantly affect the total repetition time unless t_m is reduced by about two orders of magnitude [31].

SUPPLEMENTARY REFERENCES

[1] D. T. C. Allcock, W. C. Campbell, J. Chiaverini, I. L. Chuang, E. R. Hudson, I. D. Moore, A. Ransford, C. Roman, J. M. Sage, and D. J. Wineland, Applied Physics Letters **119**, 214002 (2021).

[2] H.-X. Yang, J.-Y. Ma, Y.-K. Wu, Y. Wang, M.-M. Cao, W.-X. Guo, Y.-Y. Huang, L. Feng, Z.-C. Zhou, and L.-M. Duan, arXiv:2106.14906 [physics, physics:quant-ph] (2021), arXiv:2106.14906 [physics, physics:quant-ph].

[3] D. R. Bates and A. Damgaard, Phil. Trans. R. Soc. Lond. A **242**, 101 (1949).

[4] S. Weber, C. Tresp, H. Menke, A. Urvoy, O. Firstenberg, H. P. Büchler, and S. Hofferberth, Journal of Physics B: Atomic, Molecular and Optical Physics **50**, 133001 (2017).

[5] J. T. Wilson, S. Saskin, Y. Meng, S. Ma, R. Dilip, A. P. Burgers, and J. D. Thompson, Physical Review Letters **128**, 033201 (2022).

[6] M. Saffman, T. G. Walker, and K. Mølmer, Reviews Of Modern Physics **82**, 2313 (2010).

[7] M. J. Martin, *Quantum Metrology and Many-Body Physics: Pushing the Frontier of the Optical Lattice Clock*, Ph.D. thesis, University of Colorado, Boulder, CO (2013).

[8] S. G. Porsev, Y. G. Rakhlina, and M. G. Kozlov, Phys. Rev. A **60**, 2781 (1999).

[9] C. L. Vaillant, M. P. A. Jones, and R. M. Potvliege, Journal Of Physics B-Atomic Molecular And Optical Physics **47**, 155001 (2014).

[10] M. Aymar, R. J. Champeau, C. Delsart, and O. Robaux, Journal of Physics B: Atomic and Molecular Physics **17**, 3645 (1984).

[11] S. Ma, A. P. Burgers, G. Liu, J. Wilson, B. Zhang, and J. D. Thompson, Physical Review X **12**, 021028 (2022).

[12] S. Saskin, J. T. Wilson, B. Grinkemeyer, and J. D. Thompson, Physical Review Letters **122**, 143002 (2019).

[13] A. Bergschneider, V. M. Klinkhamer, J. H. Becher, R. Klemt, G. Zürn, P. M. Preiss, and S. Jochim, Physical Review A **97**, 063613 (2018).

[14] M. A. Joffe, W. Ketterle, A. Martin, and D. E. Pritchard, Journal of the Optical Society of America B **10**, 2257 (1993).

[15] P. McQuillen, X. Zhang, T. Strickler, F. B. Dunning, and T. C. Killian, Physical Review A **87**, 013407 (2013).

[16] T. Graham, Y. Song, J. Scott, C. Poole, L. Phuttitarn, K. Jooya, P. Eichler, X. Jiang, A. Marra, B. Grinkemeyer, *et al.*, arXiv preprint arXiv:2112.14589 (2021).

[17] R. Noek, G. Vrijsen, D. Gaultney, E. Mount, T. Kim, P. Maunz, and J. Kim, Optics Letters **38**, 4735 (2013).

[18] M. D. Lindsay, L.-T. Cai, G. W. Schinn, C.-J. Dai, and T. F. Gallagher, Physical Review A **45**, 231 (1992).

[19] W. Sandner, R. Kachru, K. A. Safinya, F. Gounand, W. E. Cooke, and T. F. Gallagher, Physical Review A **27**, 1717 (1983).

[20] H. Levine, A. Keesling, G. Semeghini, A. Omran, T. T. Wang, S. Ebadi, H. Bernien, M. Greiner, V. Vuletić, H. Pichler, and M. D. Lukin, Physical Review Letters **123**, 170503 (2019).

[21] M. B. Plenio and P. L. Knight, Reviews of Modern Physics **70**, 101 (1998).

[22] K. Mølmer, Y. Castin, and J. Dalibard, Journal of the Optical Society of America B **10**, 524 (1993).

[23] A. P. Burgers, S. Ma, S. Saskin, J. Wilson, M. A. Alarcón, C. H. Greene, and J. D. Thompson, PRX Quantum **3**, 020326 (2022).

[24] M. Saffman, I. I. Beterov, A. Dalal, E. J. Páez, and B. C. Sanders, Physical Review A **101**, 062309 (2020).

[25] D. Bluvstein, H. Levine, G. Semeghini, T. T. Wang, S. Ebadi, M. Kalinowski, A. Keesling, N. Maskara, H. Pichler, M. Greiner, V. Vuletić, and M. D. Lukin, Nature **604**, 451 (2022).

[26] S. Dörscher, R. Schwarz, A. Al-Masoudi, S. Falke, U. Sterr, and C. Lisdat, Physical Review A **97**, 063419 (2018).

[27] R. Ozeri, W. M. Itano, R. B. Blakestad, J. Britton, J. Chiaverini, J. D. Jost, C. Langer, D. Leibfried, R. Reichle, S. Seidelin, J. H. Wesenberg, and D. J. Wineland, Physical Review A **75**, 042329 (2007).

[28] G. Wolfowicz and J. J. Morton, in *eMagRes*, Vol. 5, edited by R. K. Harris and R. L. Wasylishen (John Wiley & Sons, Ltd, Chichester, UK, 2016) pp. 1515–1528.

[29] D. Wenzheng, F. Zhuang, S. Economou, and E. Barnes, PRX Quantum **2**, 030333 (2021).

[30] T. M. Graham, M. Kwon, B. Grinkemeyer, Z. Marra, X. Jiang, M. T. Lichtman, Y. Sun, M. Ebert, and M. Saffman, Physical Review Letters **123**, 230501 (2019).

[31] W. Xu, A. V. Venkatramani, S. H. Cantú, T. Šumarac, V. Klüsener, M. D. Lukin, and V. Vuletić, Physical Review Letters **127**, 050501 (2021).
